# The serum lipid profiles in immune thrombocytopenia: Mendelian randomization analysis and a retrospective study

**DOI:** 10.1186/s12959-023-00551-x

**Published:** 2023-10-13

**Authors:** Pengcheng Xu, Shouqing Han, Ming Hou, Yajing Zhao, Miao Xu

**Affiliations:** 1 Department of Hematology, Qilu Hospital, Cheeloo College of Medicine, Shandong University, No. 44 Wenhuaxi Road, Jinan, China; 2 Center for Tumor Diagnosis & Therapy, Jinshan Hospital, Fudan University, Shanghai, China; 3 Shandong Provincial Key Laboratory of Immunohematology, Cheeloo College of Medicine, Qilu Hospital, Shandong University, Shanghai, China; 4 Leading Research Group of Scientific Innovation, Department of Science and Technology of Shandong Province, Cheeloo College of Medicine, Qilu Hospital, Shandong University, Jinan, China

**Keywords:** Lipids, Platelets indices, Hemorrhage, Thrombocytopenia, Mendelian randomization

## Abstract

**Background:**

Immune thrombocytopenia (ITP) is an autoimmune hemorrhagic disease characterized by increased platelet destruction and impaired thrombopoiesis. The changes in platelet indices depend on the morphology and volume of platelets. Serum lipids have been found to affect platelet formation and activity in certain diseases, thus inducing the corresponding variation of platelet indices.

**Methods:**

Mendelian randomization (MR) analysis was performed based on databases. The clinical data from 457 ITP patients were retrospectively collected and analyzed, including platelet indices, serum lipids, hemorrhages and therapeutic responses.

**Results:**

MR analysis showed low high-density-lipoprotein-cholesterol (HDL-C), low apolipoprotein A-1, high triglyceride (TG) and high apolipoprotein B (ApoB) caused high platelet distribution width (PDW); high low-density-lipoprotein-cholesterol (LDL-C) increased mean platelet volume (MPV). In ITP, there were positive correlations between platelet count with TG, PDW with HDL-C and ApoB, and plateletcrit with TG and non-esterified fatty acid, and the correlation had gender differences. Bleeding scores were negatively correlated with cholesterol and LDL-C. LDL-C and homocysteine were risk factors for therapeutic responses.

**Conclusions:**

Serum lipids, especially cholesterol were tightly correlated with platelet indices, hemorrhage and therapeutic effects in ITP patients. These results provide clinical references for the management of serum lipids, and highlight the necessity to further explore the relationship between lipids and pathogenesis of ITP.

**Trial registration:**

No: NCT05095896, October 14, 2021, retrospectively registered.

**Supplementary Information:**

The online version contains supplementary material available at 10.1186/s12959-023-00551-x.

## Introduction

Immune thrombocytopenia (ITP) is an acquired autoimmune hemorrhagic disease characterized by decreased platelet count and abnormal platelet function [1]. The pathogenesis of ITP is heterogenous and complicated, including the generation of pathogenic anti-platelet antibodies, T cell-mediated platelet destruction and impaired platelet production [2].

Lipids are typical small hydrophobic molecules existing in all cell types. Growing evidence showed that the production and function of platelets could be dramatically affected by lipids [3]. Global lipidomic analysis has identified hundreds of lipid species during platelet coagulation [4]. Studies also found that degranulation and membrane deformation during platelet activation require membrane lipid rearrangement and fluidity [5]. Furthermore, cellular cholesterol homeostasis is well proven to be an important factor in regulating hematopoiesis, in which defective cholesterol efflux pathways could dampen thrombopoiesis [6]. Thus, it is plausible to infer that lipid profiles could influence the platelet indices, and be related to bleeding symptoms and therapeutic effects in ITP.

Platelet indices are a group of platelet parameters, commonly including mean platelet volume (MPV), platelet distribution width (PDW) and plateletcrit (PCT). Platelet indices could reflect the activated state of platelets because of the variation in morphology and volume during platelet activation [7]. Previous studies have shown that the changes in platelet indices can be used to identify thrombocytopenia caused by multiple factors and be considered as prognostic markers of disease recurrence and treatment efficacy in ITP [8, 9].

Mendelian randomization (MR) analysis is an effective method to estimate the causal effects of exposure on outcomes of disease through genome-wide association study (GWAS) data set in epidemiological studies. Compared with traditional cross-sectional observation studies, MR analysis can avoid confounding factors and reverse causality [10]. However, MR analysis could be biased if genetic variants are pleiotropic [10]. And because multiple variants are often combined into a polygenic risk score to increase statistical power, statistical power is another limitation for MR analysis [10].

In this study, we performed a bidirectional two-sample MR to comprehensively evaluate the causal relationships between serum lipids and platelet indices in the healthy population, and retrospectively analyzed the correlation among serum lipids, platelet indices and changes of platelet indices before and after treatment in ITP patients. Noticeable but inconsistent associations among lipid profile, platelet indices, bleeding symptoms and therapeutic effects were verified in healthy persons and ITP patients, indicating that serum lipids have a different influence in ITP.

## Materials and methods

### MR study design

MR analysis used the Mendelian law to allocate genetic variants randomly and instrumental variable (IV) method to identify causal effects, which could reduce confounding from environmental factors and address the issue of confounding or reverse causality [11, 12]. IVs have associations with the exposure, but they were not associated with any confounder of the exposure-outcome association, nor were there any causal pathway from the instrumental variable directly to the outcome other than via the exposure [13]. Under the assumption that a single instrumental variable or a set of instrumental variables for the exposure is available, the causal effect of the exposure on the outcome could be estimated [13]. Single nucleotide polymorphisms (SNPs), also known as genetic variants, were used as IV in MR analysis.

According to the assumptions of IV, MR analyses derived valid estimates where the following assumptions are met: [14] (i) the SNPs were associated with the exposure, (ii) the SNPs have effects on the outcome only through the exposure, and (iii) the SNPs were independent of any confounders of the exposure-outcome association. For assumption (i), the GWAS summaries of exposure and outcomes are both extracted from a database of the European population.

### Data sources

GWAS summaries for circulating lipoprotein lipids and apolipoproteins, including low density lipoprotein cholesterol (LDL-C), apolipoprotein B (ApoB), triglycerides (TG), high density lipoprotein cholesterol (HDL-C), and apolipoprotein A-1 (ApoA1) were extracted from Integrative Epidemiology Unit (IEU) database [15]. The GWAS summaries of the outcomes about platelet properties were extracted from a GWAS analysis in the UK Biobank and INTERVAL studies, which included 173,480 European-ancestry participants [16].

### Selection of genetic instruments

All relevant SNPs as genetic instruments in each dataset met the genome-wide significance threshold of the P value 5 $\times$ 10^− 8^. We performed linkage disequilibrium (LD) clumping to identify independent SNPs (r2 < 0.001 within 10 Mb), using the 1000 Genomes Project Phase 3 (EUR) as the reference panel. We harmonized exposure and outcome datasets, obtained SNP effects and corresponding standard errors, and removed palindromic SNPs with intermediate allele frequencies. We used proxy SNPs (LD at R^2^ > 0.8) when no SNP was available for predicting a specific protein for the outcome. Two parameters, the proportion of variance (R^2^) explained by the SNPs and the F statistic, were used to evaluate the instrument strength for exposures [17]. Typically, an F statistic > 10 was considered sufficiently informative for MR analyses. F statistics were calculated as the following formulae: [18]

$$F = \frac{N-k-1}{k}\times \frac{{R}^{2}}{1-{R}^{2}}$$

*R*^2^ is the proportion of the variance explained by selected instruments, N is the sample size of the GWAS for the exposure, and k is the number of selected instruments (SNPs).

### MR statistical analysis

The inverse variance-weighted (IVW) method was used as the primary MR analysis. The IVW method and the MR-Egger method were used to evaluate the heterogeneity. When the heterogeneity was high, the weighted median method and the IVW (multiplicative random effects [MRE]) method were used to provide a consistent estimate [19]. We tested for pleiotropy by performing the MR-Egger intercept test, MR pleiotropy residual sum and outlier (MR-PRESSO). All analyses were carried out using the TwoSampleMR package in R Software 4.2.3 [20]. A threshold of *P* < 0.05 was adopted to declare a statistical significance.

### Study population

A retrospective cohort study was performed at Department of Hematology of Qilu Hospital of Shandong University from January 2018 to December 2022. A total of 457 ITP patients were enrolled in this study based on international consensus of ITP [1]. Patients were excluded as (1) receiving platelet transfusion within 7 days or ITP-related medication within 3 months (2) treated with lipid-lowering agents, including statins, fibrates, niacin, bile sequestrant resins, ezetimibe, etc. This study has been approved by the ethics committee of Qilu hospital of Shandong University.

### Data collection

The basic data of the patients were all from the electronic medical record system of Qilu hospital of Shandong university. The bleeding symptoms were evaluated and recorded according to bleeding scoring system [1]. Therapeutic response was evaluated after one month of the initial treatment and “responses” were defined as a platelet count > 30 × 10^9^/L or a 2-fold increment without bleeding after treatment. The lipid samples are measured by Cobas® 8000 automated analyzer. Fresh peripheral blood from enrolled individuals was collected in a vacutainer with coagulant and separating gel. After natural coagulation, the sample was centrifuged at 1500 rpm for 15 min and the serum was separated for testing.

### Statistical analysis

All statistical analyses were performed using SPSS 25. Inter-group comparisons were analyzed by Student’s *t* test or Chi-square test (*χ*^2^ test). Correlation analyses were performed by Pearson correlation analysis, Spearman correlation analysis or Kendall correlation analysis based on normality test. Pearson correlation analysis is commonly employed when analyzing data that follows a normal distribution and involves continuous variables. Spearman correlation analysis is used for examining the relationship between a categorical variable and a continuous variable that does not adhere to a normal distribution, or when both variables are continuous but do not follow a normal distribution. Risk factors for hemorrhages and response were analyzed by univariable and multivariable logistic regression. *P* < 0.05 was considered as significant difference.

## Results

### Mendelian randomization

**Fig. 1:**
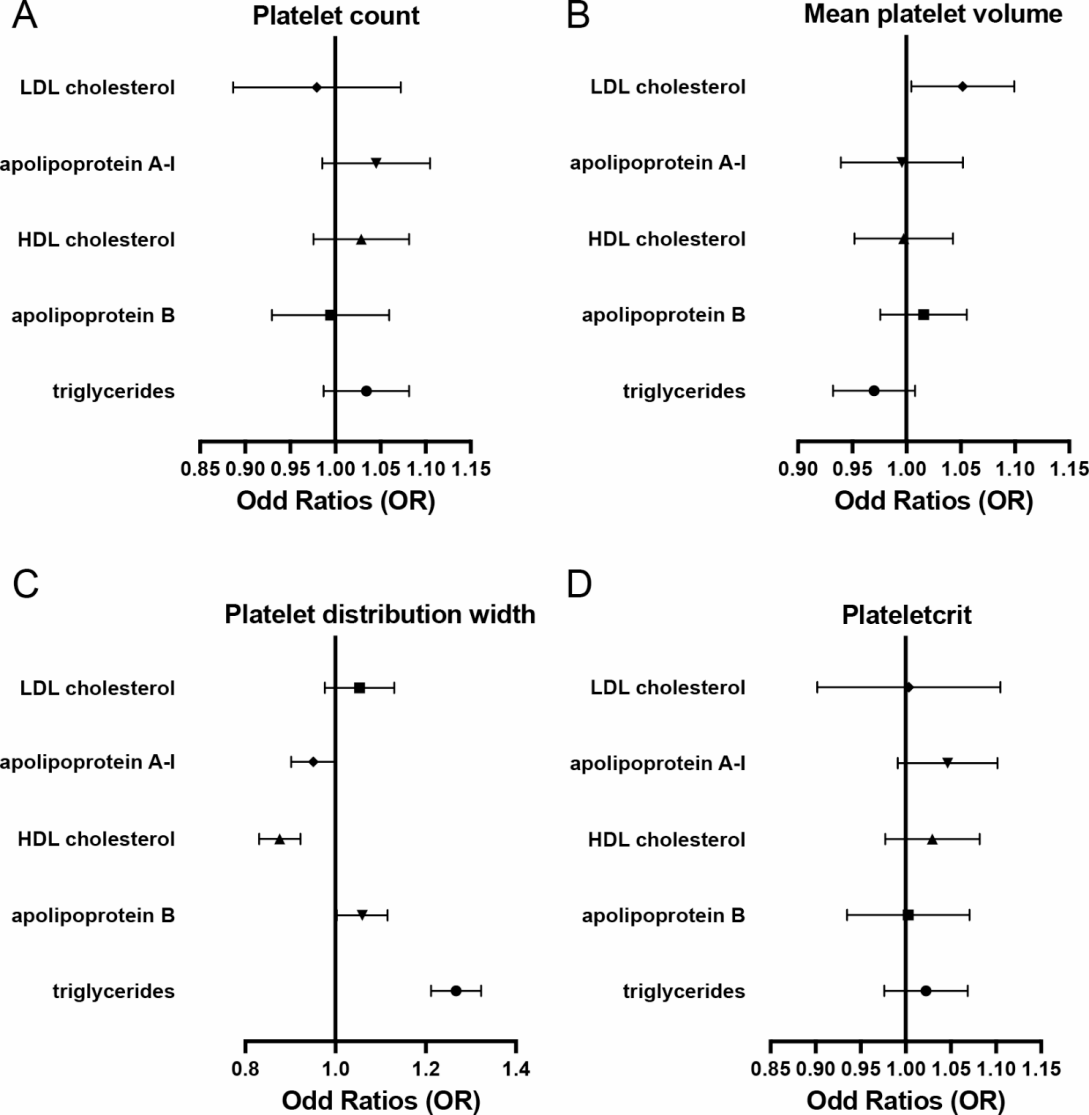
Forest plot of primary Mendelian randomization analysis. The Mendelian randomization analysis for five circulating lipoprotein lipids on four platelet indices **(A-D)** Forest plot about the causal effect of circulating lipoprotein lipids on platelet count **(A)**, mean platelet volume **(B)**, platelet distribution width **(C)** and plateletcrit **(D)**

Primary MR analysis of five circulating lipoprotein lipids and apolipoproteins on four platelet properties (Fig. 1) showed that five causal associations were significant: low HDL-C (IVW, Odds Ratio [OR], 0.88; 95% CI: 0.83–0.92, *P* < 0.01), low ApoA1 (IVW, Odds Ratio [OR], 0.95; 95% CI: 0.9-1, *P* = 0.05), high TG (IVW, Odds Ratio [OR], 1.27; 95% CI: 1.21–1.32, *P* < 0.01) and high ApoB (IVW, Odds Ratio [OR], 1.06; 95% CI: 1-1.12, *P* = 0.04) causing high PDW; and high LDL-C increasing MPV (IVW, Odds Ratio [OR], 1.05; 95% CI: 1-1.1, *P* = 0.03). (Fig. 1; Table 1)

**Table 1:** Results of MR analysis of five lipids on platelet properties

outcome	exposure	Inverse variance weighted		MR Egger		Weighted median		Inverse variance weighted (multiplicative random effects)	
		OR	*P*	OR	*P*	OR	*P*	OR	*P*
PDW	ApoA1	0.95 (0.9, 1)	0.04992471	1 (0.93, 1.09)	0.93092994	0.95 (0.91, 1)	0.03930573	0.95 (0.9, 1)	0.050
PDW	ApoB	1.06 (1, 1.12)	0.03681477	1.04 (0.97, 1.11)	0.31256384	1.05 (1.01, 1.09)	0.0142602	1.06 (1, 1.12)	0.037
PDW	HDL-C	0.88 (0.83, 0.92)	6.7162E-07	0.86 (0.8, 0.93)	0.00025656	0.93 (0.9, 0.98)	0.00225869	0.88 (0.83, 0.92)	< 0.001
PDW	triglycerides	1.27 (1.21, 1.32)	6.3416E-26	1.32 (1.24, 1.41)	4.0662E-14	1.29 (1.23, 1.35)	7.1012E-29	1.27 (1.21, 1.32)	< 0.001
MPV	LDL-C	1.05 (1, 1.1)	0.02980394	1.06 (0.99, 1.13)	0.12709535	1.07 (1.03, 1.11)	0.00031889	1.05 (1, 1.1)	< 0.001

MR: Mendelian randomization, PDW: Platelet distribution width, MPV: Mean platelet volume, ApoA1: apolipoprotein A-1, ApoB: apolipoprotein B, HDL-C: high-density-lipoprotein cholesterol, LDL-C: low-density-lipoprotein cholesterol

The IVW method and the MR-Egger method were used to evaluate the heterogeneity, and substantial heterogeneity was observed in all significant causal associations (IVW and MR-Egger, all *P* < 0.05, Table 2). However, the weighted median method and the IVW-MRE method showed the results were consistent (Table 1). All intercept estimated from the MR-Egger regression was centered on zero, and did not provide strong evidence of horizontal pleiotropy (MR-Egger, All *P* > 0.05, Table 3). However, pleiotropy was found by MR-PRESSO in all significant causal associations (All global test *P* < 0.05, Table 3), and after outlier-correction, all significant causal associations were still established (All outlier-corrected *P* < 0.05, Table 3).

**Table 2:** Heterogeneity analysis

outcome	exposure	Inverse variance weighted			MR Egger		
		Q	Q_df	Q_pval	Q	Q_df	Q_pval
PDW	ApoA1	752.3963	138	< 0.001	735.1540	137	< 0.001
PDW	ApoB	482.4643	80	< 0.001	475.6863	79	< 0.001
PDW	HDL-C	815.3041	146	< 0.001	813.3866	145	< 0.001
PDW	triglycerides	421.5132	134	< 0.001	412.3093	133	< 0.001
MPV	LDL-C	705.8174	152	< 0.001	705.7214	151	< 0.001

PDW: Platelet distribution width, MPV: Mean platelet volume, ApoA1: apolipoprotein A-1, ApoB: apolipoprotein B, HDL-C: high-density-lipoprotein cholesterol, LDL-C: low-density-lipoprotein cholesterol

**Table 3:** Pleiotropy analysis

outcome	exposure	MR-Egger		MR-PRESSO		
		egger_intercept	*P*-value	Raw *P*-value	Outlier-corrected *P*-value	Global Test *P*-value
PDW	ApoA1	-0.0023889	0.07525349	< 0.001	< 0.001	< 0.001
PDW	ApoB	0.00182644	0.29193778	0.040	0.005	< 0.002
PDW	HDL-C	0.00078889	0.55968876	< 0.001	< 0.001	< 0.003
PDW	triglycerides	-0.0017449	0.08720377	< 0.001	< 0.001	< 0.004
MPV	LDL-C	-0.0001743	0.88624577	0.067	0.002	< 0.005

PDW: Platelet distribution width, MPV: Mean platelet volume, ApoA1: apolipoprotein A-1, ApoB: apolipoprotein B, HDL-C: high-density-lipoprotein cholesterol, LDL-C: low-density-lipoprotein cholesterol

### Baseline characteristics of ITP patients

A total of 457 patients (261 females and 196 males; 18–85 years old with a median age of 47 years) were enrolled in this study (Table 4). Considering the lipid profiles, levels of total cholesterol (Cho) (4.75 ± 1.10 vs4.35 ± 1.27, *P =* 0.001), HDL-C (1.25 ± 0.37vs 1.04 ± 0.31, *P* < 0.001), ApoA1 (1.38 ± 0.29vs 1.26 ± 0.29, *P =* 0.031) and non-esterified fatty acid (NEFA) (54.90 ± 39.99 vs46.30 ± 22.77, *P =* 0.012) in women were significantly higher than those in men. Homocysteine (Hcy) (17.90 ± 8.63 vs. 12.12 ± 5.74, *P* < 0.001) in women was lower than that in men. No difference was identified in platelet indices among acute, persistent and chronic ITP patients (Supplemental Table 1).

**Table 4:** Baseline clinical and laboratory characteristics of participants

	Total (N)	male (N)	Female (N)	*P*
Age(years) ^*t*^	47.10 ± 18.13(457)	47.60 ± 19.97(196)	46.70 ± 16.63(261)	0.594
Platelet indices ^*t*^				
PC (×10^9^/L)	29.5 ± 44.48(449)	29.0 ± 42.90(193)	29.9 ± 45.72(256)	0.822
PDW (fL)	15.65 ± 3.24(167)	15.31 ± 3.29(77)	15.94 ± 3.19(90)	0.213
MPV (fL)	11.93 ± 1.84(167)	11.69 ± 1.72(77)	12.13 ± 1.93(90)	0.124
PCT (%)	0.06 ± 0.06(167)	0.06 ± 0.06(77)	0.07 ± 0.07(90)	0.574
Lipid profile ^*t*^				
**Cho (mmol/L)**	**4.58 ± 1.19(407)**	**4.35 ± 1.27(170)**	**4.75 ± 1.10(237)**	**0.001** ^**^
**HDL-C (mmol/L)**	**1.16 ± 0.36(406)**	**1.04 ± 0.31(169)**	**1.25 ± 0.37(237)**	< 0.001**
LDL-C (mmol/L)	2.76 ± 0.93(406)	2.68 ± 1.04(169)	2.82 ± 0.83(237)	0.128
sdLDL (mmol/L)	0.83 ± 0.47(398)	0.87 ± 0.47(165)	0.80 ± 0.46(233)	0.171
**ApoA1(mmol/L)**	**1.33 ± 0.29(106)**	**1.26 ± 0.29(45)**	**1.38 ± 0.29(61)**	**0.031** ^*^
ApoB (mmol/L)	1.01 ± 0.30(111)	0.95 ± 0.27(47)	1.04 ± 0.32(64)	0.128
TG (mmol/L)	1.69 ± 2.49(408)	1.69 ± 1.18(171)	1.68 ± 3.11(237)	0.975
LPA (mmol/L)	39.0 ± 53.86(105)	29.72 ± 31.07(45)	46.11 ± 65.39(60)	0.092
**Hcy (mmol/L)**	**14.54 ± 7.64(403)**	**17.90 ± 8.63(169)**	**12.12 ± 5.74(234)**	< 0.001**
**NEFA (mmol/L)**	**51.30 ± 34.12(405)**	**46.30 ± 22.77(169)**	**54.90 ± 39.99(236)**	**0.012** ^*^
PLA2(mmol/L)	569.0 ± 120.25(49)	565.0 ± 137.43(25)	573.0 ± 102.16(24)	0.826

Data were shown as mean ± standard deviation (number of cases) or positive percentage (number of positive cases / total cases).

PC: platelet count, PDW: platelet distribution width, MPV: mean platelet volume, PCT: plateletcrit, Cho: cholesterol, HDL-C: high-density-lipoprotein cholesterol, LDL-C: low-density-lipoprotein cholesterol, sdLDL: small dense low-density-lipoprotein, ApoA1: apolipoprotein A-1, ApoB: apolipoprotein B, TG: triglyceride, LPA: lipoprotein A, Hcy: homocysteine, NEFA: non-esterified fatty acid, PLA2: phospholipase A2.

^*t*^ student’s *t* test, ^*χ*^*χ*2 test.

^*^P < 0.05, ^**^ P < 0.01; *P* < 0.05 as significant difference, shown in bold font.

### Correlation analysis of serum lipid profile and platelet indices

Correlation analysis showed positive correlations between platelet count with TG (*r =* 0.195, *P* = 0.0001), PDW with HDL-C and ApoB (*r =* 0.178, *P* = 0.028; *r =* 0.386, *P* = 0.007), and PCT with TG and NEFA (*r =* 0.213, *P* = 0.008; *r =* 0.238, *P* = 0.003).

Considering the significant differences between genders of certain lipid components, the correlation analysis was performed after grouping by gender. In males, there were negative correlations between PDW and Hcy (*r =* -0.245, *P =* 0.041), MPV and phospholipase A2 (PLA2) (*r =* -0.606, *P =* 0.022), PCT and HDL-C (*r =* -0.244, *P =* 0.042), and positive correlation between PCT and NEFA (*r =* 0.276, *P =* 0.021). In females, there are positive correlations between PC and TG (*r* = 0.272, *P* < 0.001), and PCT and TG (*r* = 0.291, *P* = 0.007) (Table 5).

**Table 5:** Correlation analysis of baseline platelet indices and serum lipid profile

		PC			PDW				MPV				PCT			
		Total	Male	Female	Total		Male	Female		Total	Male	Female		Total	Male	Female
Cho	r	-0.021^P^	-0.028^P^	0.035^P^	0.099^P^		0.079^ S^	0.189^ S^		0.066^ S^	0.049^ S^	0.031^ S^		-0.086^P^	-0.111^P^	-0.058^ S^
	p	0.673	0.718	0.597	0.221		0.514	0.085		0.416	0.685	0.778		0.289	0.359	0.598
HDL-C	r	-0.036^P^	-0.062^P^	-0.021^P^	**0.178** ^*P^		0.215^ S^	0.164^P^		0.094^ S^	0.197^ S^	0.024^ S^		-0.066^P^	**-0.244** ^*P^	0.019^P^
	p	0.466	0.424	0.743	**0.028**		0.074	0.135		0.244	0.102	0.827		0.413	**0.042**	0.866
LDL-C	r	-0.030^P^	-0.026^P^	-0.035^P^	0.059^P^		-0.009^ S^	0.093^P^		0.077^ S^	0.053^ S^	0.082^ S^		-0.079^P^	-0.062^P^	-0.101^P^
	p	0.543	0.743	0.594	0.468		0.943	0.403		0.343	0.666	0.458		0.329	0.613	0.359
sdLDL	r	0.005^P^	0.063^P^	-0.039^P^	-0.023^P^		-0.078^ S^	0.051^P^		-0.015^ S^	-0.063^ S^	0.043^ S^		-0.066^P^	0.040^P^	-0.148^P^
	p	0.925	0.426	0.551	0.778		0.521	0.649		0.858	0.603	0.697		0.420	0.740	0.182
APOA1	r	-0.052^ S^	-0.047^ S^	-0.047^ S^	-0.042^ S^		0.102	-0.126^ S^		-0.216^ S^	-0.136^ S^	-0.158^ S^		-0.064^ S^	-0.257^ S^	-0.072^ S^
	p	0.596	0.759	0.722	0.782		0.643	0.576		0.154	0.537	0.484		0.675	0.236	0.751
APOB	r	0.156^ S^	0.002^P^	0.220^ S^	**0.386** ^**S^		0.366^ S^	0.357^ S^		0.113^ S^	-0.116^ S^	0.190^ S^		0.050^ S^	-0.076^P^	0.097^ S^
	p	0.101	0.990	0.080	**0.007**		0.079	0.095		0.449	0.590	0.386		0.740	0.723	0.661
TG	r	**0.195** ^**P^	0.019^P^	**0.272** ^**P^	-0.05^P^		-0.038^ S^	-0.069^P^		-0.033^ S^	-0.076^ S^	0.006^ S^		**0.213** ^**P^	0.008^P^	**0.291** ^**P^
	p	**< 0.001**	0.808	**< 0.001**	0.538		0.757	0.531		0.685	0.530	0.956		**0.008**	0.949	**0.007**
LPA	r	-0.001^P^	-0.095^P^	0.037^P^	0.187^P^		-0.149^ S^	0.177^P^		0.082^ S^	0.164^ S^	-0.009^ S^		-0.126^P^	-0.167^P^	-0.203^P^
	p	0.995	0.533	0.776	0.224		0.496	0.444		0.598	0.454	0.969		0.414	0.448	0.377
Hcy	r	0.056^P^	0.067^P^	0.043^P^	-0.091^P^		**-0.245** ^*S^	0.067^P^		-0.130	-0.136^ S^	-0.093^ S^		0.060^P^	0.096^P^	0.040^P^
	p	0.264	0.389	0.516	0.265		**0.041**	0.546		0.109	0.260	0.405		0.459	0.430	0.722
NEFA	r	0.029^P^	0.112^P^	-0.004^P^	0.031^P^		0.025^ S^	-0.023^P^		0.108^ S^	0.140^ S^	0.091^ S^		**0.238** ^**P^	**0.276** ^*P^	0.199^P^
	p	0.564	0.151	0.951	0.700		0.84	0.839		0.185	0.249	0.415		**0.003**	**0.021**	0.071
PLA2	r	0.105^ S^	0.102^P^	0.144^ S^	0.393^ S^		0.319^P^	0.418^ S^		-0.005^ S^	**-0.606** ^*S^	0.328^ S^		0.016^ S^	0.030^P^	-0.150^ S^
	p	0.472	0.629	0.502	0.052		0.267	0.201		0.981	**0.022**	0.325		0.939	0.920	0.659

PC: platelet count, PDW: Platelet distribution width, MPV: Mean platelet volume, PCT: plateletcrit, Cho: cholesterol, HDL-C: high-density-lipoprotein cholesterol, LDL-C: low-density-lipoprotein cholesterol, ApoA1: apolipoprotein A-1, ApoB: apolipoprotein B, TG: triglyceride, LPA: lipoprotein A, Hcy: homocysteine, NEFA: non-esterified fatty acid, PLA2: phospholipase A2.

^S^ Spearman correlation analysis; ^P^ Pearson correlation analysis.

^*^P < 0.05, ^**^ P < 0.01; *P* < 0.05 as significant difference, shown in bold font.

### Correlation analysis and risk factors for hemorrhagic symptoms

Hemorrhagic symptoms were quantified as bleeding scores and correlation analysis results showed that bleeding scores were negatively correlated with Cho and LDL-C (*r* = -0.320, *P =* 0.014; *r* = -0.354, *P =* 0.006) (Table 6). Regression analysis showed that Cho (*P =* 0.008), LDL-C (*P =* 0.001) and sdLDL-C (*P =* 0.041) were significant factors accounting for hemorrhages based only on univariate analysis, while LDL-C was significant in multivariate regression (*P* = 0.01) (Table 7).

**Table 6:** Correlation ananlysis of bleeding score

		PC	PDW	MPV	PCT	Cho
Bleeding score	*r*	-0.004	-0.141	-0.142	0.012	**-0.320** ^*^
	*P*	0.976	0.502	0.501	0.955	**0.014**
		HDL-C	LDL-C	sdLDL	ApoA1	ApoB
	*r*	-0.191	**-0.354** ^**^	-0.125	-0.098	0.074
	*P*	0.142	**0.006**	0.335	0.761	0.802
		TG	LPA	Hcy	NEFA	PLA2
	*r*	-0.027	-0.293	-0.166	-0.124	-0.15
	*P*	0.837	0.362	0.2	0.343	0.645

PC: platelet count, PDW: Platelet distribution width, MPV: Mean platelet volume, PCT: plateletcrit, Cho: cholesterol, HDL-C: high-density-lipoprotein cholesterol, LDL-C: low-density-lipoprotein cholesterol, sdLDL: small dense low-density-lipoprotein, ApoA1: apolipoprotein A-1, ApoB: apolipoprotein B, TG: triglyceride, LPA: lipoprotein A, Hcy: homocysteine, NEFA: non-esterified fatty acid, PLA2: phospholipase A2

^*^P < 0.05, ^**^ P < 0.01; *P* < 0.05 as statistical significance, shown in bold font

All results were performed by Spearman correlation analysis

**Table 7:** Regression analysis of risk factors for hemorrhages and response

Outcome	Variable	Univariate HR (95% CI)	*P*	Multivariate HR (95% CI)	*P*	Percentage Correct	Overall Percentage Correct
Hemorrhages	Cho	0.599 (0.409–0.876)	0.008	1.294 (0.657–2.549)	0.46	76.7	78.6
	LDL-C	0.357 (0.198–0.643)	0.001	0.267 (0.104–0.686)	0.01	75.9	
	sdLDL	0.341 (0.121–0.956)	0.041	0.982 (0.247–3.901)	0.98	76.6	
Response	LDL-C	0.633 (0.445–0.881)	0.007	0.627 (0.447–0.879)	0.01	66.7	68.4
	Hcy	0.954 (0.914–0.995)	0.027	0.954 (0.913–0.995)	0.03	68.9	

Cho: cholesterol, LDL-C: low-density-lipoprotein cholesterol, sdLDL: small dense low-

density-lipoprotein, Hcy: homocysteine

### Regression analysis of risk factors for therapeutic response

A total of 198 patients with one month follow-up data were enrolled in regression analysis. For therapeutic response, LDL-C and Hcy were both significant as risk factors in univariate (*P =* 0.007, *P =* 0.027) and multivariate regression analysis (*P =* 0.01, *P =* 0.03) (Table 7).

## Discussion

Lipids have been reported to impinge on platelet activation and immune responses in autoimmune diseases. However, the role of lipid profiles in ITP remains to be investigated. Our results emphasize the relationship of lipids and platelet indices, verify the importance of serum lipids in ITP and found the correlations of lipids with platelet indices, hemorrhagic symptoms and therapeutic effects.

The correlations of platelet indices with lipid profiles have been reported by several studies on normal populations and some diseases [7, 21, 22]. However, the results were not completely consistent based on demographic characteristics and disease background, suggesting the different potential mechanisms of lipids influencing platelets. Our MR analysis showed in European-ancestry participants, HDL-C and ApoA1 negatively affect PDW, while TG and ApoB positively affect PDW, but the relationship is not the same in ITP and only adapts to the European population (The large database of Chinese population is not available). We found that ApoB had a positive relationship with PDW, which is compatible with MR analysis. However, the positive correlation of PDW with HDL-C we found is unique in ITP patients in comparison to normal individuals or inflammatory diseases such as familial Mediterranean fever [7, 22]. PDW could reflect the activation status of platelets because of the variation in morphology and size during platelet activation, and commonly elevate in ITP patients. HDL, as a cholesterol receptor, can promote cholesterol outflow and participate in the activation of platelets in cardiovascular diseases [23]. Also, previous studies have reported that HDL inhibits the activation of platelets in cardiovascular events at a long-time follow-up [24, 25]. Deanna L Plubell et al. reported that the dynamic changes of HDL in the acute phase of stroke are associated with recovery [26], which may be attributed to inflammatory remodeling. The positive correlation of PDW and HDL-C in ITP would imply the regulatory potential of HDL-C in platelet status in ITP.

In this study, most platelet indices, such as PC, PDW and PCT, had considerable correlation with serum levels of TG, HDL-C, and NEFA in ITP. Among these, MPV had no significant correlation with any lipid parameters in ITP, but positively correlated with LDL-C in MR analysis based on healthy people. The increase of MPV is also a determinant of platelet activation. Several large-cohort analyses implied that high LDL-C level and high MPV index are often concomitant in infections and coronary artery diseases [27–29]. Yet, the mechanism of LDL-C’s influence on MPV is still unclear. We also found gender-wise differences of platelet indices and serum lipid profiles in ITP, which could also be found in healthy groups [22], presumably explained by (1) the differential distribution of lipids between genders, for example, higher Cho, HDL-C, ApoA1, NEFA and lower Hcy in female patients; (2) gender-different hormones, such as estradiol, which has been shown to affect thrombopoiesis [30].

Bleeding is the main manifestation in ITP, ranging from skin petechiae to life-threatening intracranial hemorrhage. Studies found the activation of platelets, independent of platelet count, was associated with bleeding severity in ITP [31]. We found Cho, LDL-C and sdLDL-C were negatively correlated with hemorrhages in our study. LDL-C could sensitize platelets and enhance the activation, aggregation and secretion response of platelets via receptor-mediated signaling cascades and lipid exchange [23]. Besides, plasma oxidized LDL-C could elevate the production of the procoagulant protein tissue factor in monocytes and endothelial cells, which also contribute to the hemostasis [23]. Thus, our study suggests that the higher levels of Cho, LDL-C and sdLDL-C might reduce hemorrhages in ITP patients due to the stronger activation of platelets.

Though higher LDL-C may be a protective factor in bleeding, we found LDL-C and Hcy are risk factors for poor therapeutic response in ITP. Overactivated immune response is the pathogenic mechanism of ITP, while restoring immune intolerance is the essential therapeutic strategy. LDL-C accumulation could induce a variety of inflammatory responses in some diseases, such as atherosclerosis [23]. Autoantigens derived from LDL-C particles can activate macrophages, promote T cells differentiating into T helper type 1 (Th1) subtype and stimulate B cells for autoantibody production [23]. On the other hand, previous studies have demonstrated that Hcy can potentially exacerbate the inflammatory response by influencing various immune cells. For instance, it has been shown to enhance reactive oxygen species (ROS) generation, activate the NLRP3 inflammasome, and induce pyroptosis in macrophages [32]. Additionally, Hcy has been implicated in activating Th1 and Th17 cells, neutrophils, and suppressing regulatory T cells, thereby promoting the production of pro-inflammatory cytokines [33]. Based on our results, the modulating of LDL-C and Hcy may be beneficial to the therapeutic response by restoring immune imbalance.

The application of lipid-lowing agents such as statins has shown to be effective in ameliorating autoimmune symptoms by targeting several immune cells, including relieving pathogenic T cell-mediated responses, reducing proinflammatory macrophages and modulating antigen presentation of dendritic cells [34]. Our previous study also showed the regulatory effects of atorvastatin on CD4^+^ T cells in ITP [35]. Despite immune modulation, statins have been found to improve thrombopoiesis by restoring impaired bone marrow endothelial progenitor cells [36]. Therefore, the importance of lipid management may be underestimated in ITP.

In general, this study showed the influence of lipids on platelet indices by MR analysis in European-ancestry participants, and provides evidence by a retrospective study in ITP that lipids may influence platelet status, bleeding and hemostasis, and immune response. These results could provide clinical references for the management of lipids in ITP and suggest that lipid regulating medicine is anticipated to be a potential adjuvant therapy for ITP.

## Conclusions

This study found that serum lipids, especially cholesterol, were tightly correlated with platelet indices in ITP patients, and cholesterol and LDL-C were negatively correlated with hemorrhage.

Furthermore, we found LDL-C and homocysteine were risk factors for therapeutic response. These results provide clinical references for the management of serum lipids, and highlight the necessity to further explore the relationship between lipids and pathogenesis of ITP.

### Electronic supplementary material

Below is the link to the electronic supplementary material.

Supplementary Material 1

## Data Availability

All data generated or analysed during this study are included in this published article.

## List of abbreviations

ApoA1: apolipoprotein A-1
ApoB: apolipoprotein B
Cho: cholesterol
GWAS: genome-wide association study
Hcy: homocysteine
HDL-C: high density lipoprotein cholesterol
IEU: Integrative Epidemiology Unit
ITP: immune thrombocytopenia
IV: instrumental variable
IVW: inverse variance-weighted
LD: linkage disequilibrium
LDLC: low density lipoprotein cholesterol
LPA: lipoprotein A
MPV: mean platelet volume
MR: Mendelian randomization
MRE: multiplicative random effects
MR-PRESSO: MR pleiotropy residual sum and outlier
NEFA: non-esterified fatty acid
PC: platelet count
PCT: plateletcrit
PDW: platelet distribution width
PLA2: phospholipase A2
sdLDL: small dense low-density-lipoprotein
SNPs: single nucleotide polymorphism
TG: triglycerides
