# Supplementary material for: The serum lipid profiles in immune thrombocytopenia: Mendelian randomization analysis and a retrospective study
Source: Thromb J. 2023 Oct 13;21:107. doi: 10.1186/s12959-023-00551-x (PMC10571271; doi:10.1186/s12959-023-00551-x)
Supplement: Supplementary file 1 — Supplementary Material 1 [file 12959_2023_551_MOESM1_ESM.docx]

| **Supplemental table 1: Platelet indices among acute, persistent and chronic ITP patients** |
| --- |

|  | Percentage | PC(×109/L) | PDW(fL) | MPV(fL) | PCT (%) |
| --- | --- | --- | --- | --- | --- |
| Acute ITP | 30.65% | 38.6±61.51 | 15.79±4.28 | 11.49±1.65 | 0.094286±0.087 |
| Persistent ITP | 25.80% | 33.6±37.78 | 14.47±2.82 | 11.75±1.31 | 0.072±0.045 |
| Chronic ITP | 43.55% | 20.77±46.65 | 16.83±4.54 | 11.48±2.24 | 0.043±0.059 |

PC: platelet count, PDW: Platelet distribution width, MPV: Mean platelet volume，PCT: plateletcrit
